# *HLA-DRB1* and *DQB1* alleles in Japanese type 1 autoimmune hepatitis: The predisposing role of the DR4/DR8 heterozygous genotype

**DOI:** 10.1371/journal.pone.0187325

**Published:** 2017-10-31

**Authors:** Shomi Oka, Hiroshi Furukawa, Michio Yasunami, Aya Kawasaki, Hitomi Nakamura, Minoru Nakamura, Atsumasa Komori, Seigo Abiru, Shinya Nagaoka, Satoru Hashimoto, Atsushi Naganuma, Noriaki Naeshiro, Kaname Yoshizawa, Haruhiro Yamashita, Keisuke Ario, Hajime Ohta, Hironori Sakai, Iwao Yabuuchi, Atsushi Takahashi, Kazumichi Abe, Hiroshi Yatsuhashi, Shigeto Tohma, Hiromasa Ohira, Naoyuki Tsuchiya, Kiyoshi Migita

**Affiliations:** 1 Molecular and Genetic Epidemiology Laboratory, Faculty of Medicine, University of Tsukuba, 1-1-1 Tennodai, Tsukuba, Japan; 2 Clinical Research Center for Allergy and Rheumatology, National Hospital Organization Sagamihara National Hospital, 18–1 Sakuradai, Minami-ku, Sagamihara, Japan; 3 Department of Medical Genomics, Life Science Institute, Saga-ken Medical Centre Koseikan, 400 Kasemachi-Nakabaru, Saga, Japan; 4 Department of Clinical Medicine, Institute of Tropical Medicine, Nagasaki University, 1-12-4 Sakamoto, Nagasaki, Japan; 5 Department of Hepatology, Nagasaki University Graduate School of Biomedical Sciences, 1-12-4 Sakamoto, Nagasaki, Japan; 6 Clinical Research Center, National Hospital Organization Nagasaki Medical Center, 2-1001-1 Kubara, Omura, Japan; 7 NHO-AIH study group, National Hospital Organization Nagasaki Medical Center, 2-1001-1 Kubara, Omura, Japan; 8 Department of Gastroenterology and Rheumatology, Fukushima Medical University School of Medicine, 1 Hikarigaoka, Fukushima, Japan; Istituto di Ricovero e Cura a Carattere Scientifico Centro di Riferimento Oncologico della Basilicata, ITALY

## Abstract

**Objective:**

Autoimmune hepatitis (AIH) is a chronic progressive liver disease. AIH is composed predominantly of type 1 in Japanese populations. The genetic and environmental factors are associated with the pathogenesis of AIH. *HLA*-DRB1*03:*01* and *04:*01* are associated with type 1 AIH in European and *04:*05* in Japanese populations. Here, we conducted an *HLA* association study in order to find *HLA* alleles or haplotypes predisposing or protective for Japanese AIH.

**Methods:**

*HLA*-*DRB1* and *DQB1* genotyping of 360 type 1 AIH patients and 1026 healthy controls was performed.

**Results:**

The predisposing association of DRB1*04:*01* (*P* = 0.0006, corrected *P* [*Pc*] = 0.0193, odds ratio [OR] 2.97, 95% confidence interval [CI] 1.62–5.43), DRB1*04:*05* (*P* = 1.89×10^−21^, *Pc* = 5.86×10^−20^, OR 3.41, 95% CI 2.65–4.38), and DQB1*04:*01* (*P* = 4.66×10^−18^, *Pc* = 6.99×10^−17^, OR 3.89, 95% CI 2.84–5.33) and the protective association of DRB1*13:*02* (*P* = 0.0003, *Pc* = 0.0080, OR 0.48, 95% CI 0.32–0.72) with Japanese type 1 AIH were observed. An association of the DR4/DR8 heterozygous genotype with Japanese AIH was identified for the first time (*P* = 3.12×10^−9^, OR 3.52, 95% CI 2.34–5.29). Susceptible diplotypes were DRB1*04:05-DQB1*04:01/DRB1*08:02-DQB1*03:*02* (*P* = 0.0004, OR 24.77, 95% CI 1.45–424.31) and DRB1*04:05-DQB1*04:01/DRB1*08:03-DQB1*06:*01* (*P* = 1.18×10^−6^, OR 10.64, 95% CI 3.19–35.46). Serum levels of Immunoglobulin G and Immunoglobulin M, International Autoimmune Hepatitis Group score, positive rate of anti-smooth muscle antibodies, and the rate of definite AIH were higher in AIH patients with DRB1*04:*05* than without.

**Conclusions:**

The important roles of specific combinations of *DRB1* and *DQB1* alleles or haplotypes in the pathogenesis of type 1 AIH were suggested. The association of DR4/DR8 heterozygous genotype suggested the pathologic importance of trans-complementing DQα-β heterodimer molecules encoded by *DQA1* allele of one haplotype and the *DQB1* allele of the other haplotype, as it was proposed in the *HLA* association studies of Type 1 diabetes.

## Introduction

Autoimmune hepatitis (AIH) is a very rare chronic progressive liver disease with autoimmune features [1,2,3]. Type 1 AIH is characterized by the presence of serum anti-nuclear antibodies (ANA) or anti-smooth muscle antibodies (ASMA) and type 2 AIH by type 1 liver-kidney microsomal antibodies. AIH is composed predominantly of type 1 in Japanese populations. Although the disease etiology is uncertain, it is considered that the genetic and environmental factors are associated with the pathogenesis of AIH. Many studies including a recent genome-wide association study [4] showed the genetic association of AIH with genes located within human leukocyte antigen (*HLA*) region. *HLA*-DRB1*03:*01* and *04:*01* are associated with AIH in European populations [5]; *04:*05* is associated in Japanese and Korean populations [6,7,8,9]. In addition, several studies have shown that DRB1*04:*04*, *04:*05*, and *13:*01* are associated with AIH in Latin America [10,11,12,13]. DRB1*08 alleles are also reported to be associated with AIH in Indian and Iranian, but not in Pakistani populations [14,15,16]. On the other hands, DRB1*15:*01* is protective for the susceptibility of AIH in European and Japanese populations [5,6]. DRB1*13:*02*, which differ by one amino acid residue from DRB1*13:*01*, is protectively associated with AIH in Latin America [11,13,17] and in Japan [8].

It was reported in the genome-wide association study that *HLA* is the sole strong genetic factor for the susceptibility of type 1 AIH [4]. The *HLA* region was scanned and the most important loci for the susceptibility of type 1 AIH was reported to be *DRB1* [18]. It was suggested that no other genes in the *HLA* region are associated with type 1 AIH. However, *DRB1* is in strong linkage disequilibrium with *DQB1* and it is difficult to differentiate the role of *DRB1* and *DQB1* in the pathogenesis of type 1 AIH. Although *HLA* alleles are known to confer the risk for various autoimmune diseases, the precise mechanisms have not sufficiently been revealed. The risk alleles are different in these autoimmune diseases [19]. It was considered that different auto-antigens are presented by different disease-specific risk alleles; the presented auto-antigens are restricted by *HLA* alleles and are influenced by non-*HLA* genes, environmental factors, or precipitating events. The complex of auto-antigens and risk alleles stimulate self-reactive T cells, resulting in the eliciting of diseases [20]. In this study, we conducted an *HLA* association study in order to search *HLA* alleles or haplotypes predisposing or protective for Japanese AIH.

## Materials and methods

### Patients and healthy controls

Three hundred sixty type 1 AIH patients were enrolled from the register of Japanese National Hospital Organization Liver Registry [21]. The AIH patients without any other types of liver diseases satisfied the criteria of International Autoimmune Hepatitis Group (IAIHG) for diagnosis of type I AIH [22]. The healthy controls (n = 1026; mean age ± SD, 37.7 ± 11.7 years, 303 male [29.8%]) were recruited at Sagamihara Hospital, the University of Tokyo, Teikyo University, and Kanazawa University [23,24] or by the Pharma SNP Consortium (Tokyo, Japan) [25]. All the patients and the healthy individuals were native Japanese living in Japan. The study was reviewed and approved by University of Tsukuba Research Ethics Committee, Nagasaki University Research Ethics Committee, and the NHO central Institutional Review Board. Informed consents in writing were obtained from all the participants. The study was performed in accordance with the principles expressed in the Declaration of Helsinki.

### Genotyping methods

Genotyping of *HLA-DRB1* and *DQB1* was conducted by the polymerase chain reaction with sequence-specific oligonucleotide probes (WAKFlow HLA typing kits, Wakunaga, Hiroshima, Japan), using the Bio-Plex 200 system (Bio-Rad, Hercules, CA). HLA-DR4 serological group includes DRB1*04:*01*, *04:*03*, *04:*04*, *04:*05*, *04:*06*, *04:*07*, and *04:*10*. DR6 is composed of DRB1*13:*01*, *13:*02*, *14:*03*, *14:*04*, *14:*05*, *14:*06*, *14:*07*, *14:*29*, and *14:*54*. DR8 consists of DRB1*08:*02*, *08:*03*, and *08:*09*. Genotyping results of *HLA-DRB1* and *DQB1* for some of the AIH patients were previously reported [8]. Genotyping results of *DRB1* for all of the healthy controls (n = 1026) were previously reported [8,23,24]. Reported genotyping results of *DQB1* for some of the healthy controls (n = 413; mean age ± SD, 39.3 ± 11.0 years, 61 male [14.8%]) were used for the analyses on *DQB1* allele, *DQB1* genotype, *DRB1-DQB1* haplotype, *DRB1-DQB1* diplotype, and acid residues in the DQβ chain [24]. *DRB1-DQB1* haplotypes were elucidated by direct counting, because *DRB1* is in strong linkage disequilibrium with *DQB1*.

### Statistical analysis

Differences of AIH characteristics were analyzed by Mann-Whitney's U test or Fisher’s exact test using 2x2 contingency tables. Association of allele carrier frequencies, haplotype carrier frequencies, or amino acid residue carrier frequencies was analyzed by Fisher’s exact test using 2x2 contingency tables under the dominant model. Differences of genotype frequencies or diplotype (the specific combination of *DRB1*-*DQB1* haplotypes) frequencies were analyzed by Fisher’s exact test using 2x2 contingency tables. Adjustment for multiple comparisons was conducted with Bonferroni method; corrected P (*Pc*) values were calculated by multiplying the *P* value by the number of alleles or amino acid residues tested.

## Results

### Clinical features of type I AIH patients

Characteristics of the type I AIH patients are shown in Table 1. Among 360 AIH patients, 314 (87.2%) were positive for ANA, 118 (38.6%) were positive for ASMA. Of overall AIH, 227 (63.1%) were definite AIH.

**Table 1:** Characteristics of type I AIH patients.

	AIH
Number	360
Male, n (%)	43 (11.9%)
Mean age, years (SD)	62.9 (±13.5)
Age at onset, years (SD)	59.1 (±13.5)
Albumin (g/dl) (SD)	3.8 (±0.6)
Total bilirubin (mg/dl) (SD)	3.7 (±4.9)
AST(IU/L) (SD)	469.7 (±546.2)
ALT(IU/L) (SD)	507.5 (±510.1)
ALP(IU/L) (SD)	465.2 (±211.0)
IgG (mg/dl) (SD)	2413.6 (±898.2)
IgM(mg/dl) (SD)	205.9 (±228.0)
Platelets (10^4^/μl) (SD)	18.7 (±7.1)
ANA ≧ 1:40, n (%)	314 (87.2%)
ASMA ≧ 1:40, n (%)	118 (38.6%)
Cirrhosis, n (%)	49 (13.6%)
IAIHG score (SD)	16.3 (±3.1)
Definite AIH, n (%)	227 (63.1%)

AIH: autoimmune hepatitis, AST: aspartate aminotransferase, ALT: alanine aminotransferase, ALP: Alkaline Phosphatase, IgG: immunoglobulin G, IgG: immunoglobulin M, ANA: anti-nuclear antibody, ASMA: anti-smooth muscle antibody, IAIHG: International Autoimmune Hepatitis Group. Numbers or average values of each group are shown. Percentages or standard deviations are shown in parenthesis.

### *HLA-DRB1* in type I AIH

To compare *HLA-DRB1* allele carrier frequency of the AIH patients and the healthy controls, we performed *HLA-DRB1* genotyping (Table 2). A significant association between type I AIH and DRB1*04:*05* (*P* = 1.89×10^−21^, corrected *P* [*Pc*] = 5.86×10^−20^, odds ratio [OR] 3.41, 95% confidence interval [CI] 2.65–4.38) was detected. DRB1*04:*01* was also associated with type I AIH (*P* = 0.0006, *Pc* = 0.0193, OR 2.97, 95%CI 1.62–5.43). On the contrary, DRB1*13:*02* was found to be protectively associated with type 1 AIH (*P* = 0.0003, *Pc* = 0.0080, OR 0.48, 95% CI 0.32–0.72). HLA-DR4 serological group was associated with type I AIH (*P* = 3.84X10^-18^, OR 2.98, 95% CI 2.32–3.83), but DR6 is protectively associated (*P* = 2.10X10^-5^, OR 0.54, 95% CI 0.41–0.72). Thus, DRB1*04:05 and DRB1*04:*01* were predisposing and DRB1*13:*02* was protective for AIH.

**Table 2:** *HLA-DRB1* allele carrier frequency in the AIH patients and healthy controls.

	Case (n = 360)	Control (n = 1026)	*P*	OR	*P*c	95%CI
DRB1*01:*01*	26 (7.2)	110 (10.7)	0.0634	0.65	NS	(0.42–1.01)
DRB1*03:*01*	1 (0.3)	3 (0.3)	1.0000	0.95	NS	(0.10–9.16)
DRB1*04:*01*	22 (6.1)	22 (2.1)	0.0006	2.97	0.0193	(1.62–5.43)
DRB1*04:*03*	15 (4.2)	47 (4.6)	0.8823	0.91	NS	(0.50–1.64)
DRB1*04:*04*	0 (0.0)	4 (0.4)	0.5780	0.32	NS	(0.02–5.87)
DRB1*04:*05*	185 (51.4)	243 (23.7)	1.89X10^-21^	3.41	5.68X10^-20^	(2.65–4.38)
DRB1*04:*06*	15 (4.2)	76 (7.4)	0.0351	0.54	NS	(0.31–0.96)
DRB1*04:*07*	5 (1.4)	15 (1.5)	1.0000	0.95	NS	(0.34–2.63)
DRB1*04:*10*	12 (3.3)	32 (3.1)	0.8616	1.07	NS	(0.55–2.10)
DRB1*07:*01*	2 (0.6)	9 (0.9)	0.7382	0.63	NS	(0.14–2.94)
DRB1*08:*02*	35 (9.7)	72 (7.0)	0.1080	1.43	NS	(0.93–2.18)
DRB1*08:*03*	58 (16.1)	153 (14.9)	0.6091	1.10	NS	(0.79–1.52)
DRB1*08:*09*	0 (0.0)	2 (0.2)	1.0000	0.57	NS	(0.03–11.87)
DRB1*09:*01*	77 (21.4)	280 (27.3)	0.0298	0.72	0.8949	(0.54–0.97)
DRB1*10:*01*	5 (1.4)	5 (0.5)	0.1381	2.88	NS	(0.83–9.99)
DRB1*11:*01*	8 (2.2)	41 (4.0)	0.1361	0.55	NS	(0.25–1.18)
DRB1*12:*01*	25 (6.9)	75 (7.3)	0.9059	0.95	NS	(0.59–1.51)
DRB1*12:*02*	11 (3.1)	37 (3.6)	0.7384	0.84	NS	(0.43–1.67)
DRB1*13:*01*	2 (0.6)	8 (0.8)	1.0000	0.71	NS	(0.15–3.36)
DRB1*13:*02*	30 (8.3)	163 (15.9)	0.0003	0.48	0.0080	(0.32–0.72)
DRB1*14:*02*	1 (0.3)	0 (0.0)	0.2597	8.57	NS	(0.35–210.76)
DRB1*14:*03*	5 (1.4)	44 (4.3)	0.0078	0.31	0.2337	(0.12–0.80)
DRB1*14:*04*	0 (0.0)	4 (0.4)	0.5780	0.32	NS	(0.02–5.87)
DRB1*14:*05*	13 (3.6)	40 (3.9)	0.8744	0.92	NS	(0.49–1.75)
DRB1*14:*06*	5 (1.4)	29 (2.8)	0.1655	0.48	NS	(0.19–1.26)
DRB1*14:*07*	1 (0.3)	2 (0.2)	1.0000	1.43	NS	(0.13–15.78)
DRB1*14:*54*	20 (5.6)	58 (5.7)	1.0000	0.98	NS	(0.58–1.66)
DRB1*15:*01*	41 (11.4)	139 (13.5)	0.3171	0.82	NS	(0.57–1.19)
DRB1*15:*02*	62 (17.2)	224 (21.8)	0.0692	0.74	NS	(0.55–1.02)
DRB1*16:*02*	5 (1.4)	18 (1.8)	0.8119	0.79	NS	(0.29–2.14)
DR4	238 (66.1)	406 (39.6)	3.84X10^-18^	2.98		(2.32–3.83)
DR6 (*13, *14)	74 (20.6)	332 (32.4)	2.10X10^-5^	0.54		(0.41–0.72)
DR8	92 (25.6)	220 (21.4)	0.1234	1.26		(0.95–1.66)

AIH: autoimmune hepatitis, OR: odds ratio, CI: confidence interval, *P*c: corrected *P* value, NS: not significant. Allele carrier frequencies are shown in parenthesis (%). Association was tested by Fisher's exact test using 2x2 contingency tables under the dominant model.

*Demographic features of type I AIH patients with or without* DRB1*04:05 *or* *13:02

Clinical features of AIH patients with or without DRB1*04:05 or *13:*02* were compared (Table 3). Serum levels of Immunoglobulin G (IgG), Immunoglobulin M (IgM), and IAIHG score were higher in AIH patients with DRB1*04:*05* than without. Positive rate of ASMA and the rate of definite AIH were higher in AIH patients with DRB1*04:*05* than without. The complication rate of cirrhosis tended to be higher in AIH patients with DRB1*13:*02* than without. Thus, specific clinical features of AIH patients possessing DRB1*04:*05* were observed.

**Table 3:** Comparison of the demographics between AIH patients with or without DRB1*04:*05* or *13:*02*.

	DRB1*04:*05*(+)	DRB1*04:*05*(-)	*P*	DRB1*13:*02*(+)	DRB1*13:*02*(-)	*P*
Number	185	175		30	330	
Male, n (%)	20 (10.8%)	23 (13.1%)	*0.5193	2 (6.7%)	41 (12.4%)	*0.5559
Age at onset, years (SD)	58.1 (±14.3)	59.2 (±14.7)	0.2897	59.5 (±15.8)	58.5 (±14.4)	0.6174
Mean age, years (SD)	63.2 (±12.2)	62.6 (±14.8)	0.6877	62.0 (±15.9)	63.0 (±13.3)	0.9408
Albumin (g/dl) (SD)	3.7 (±0.7)	3.8 (±0.9)	0.2699	3.7 (±0.6)	3.8 (±0.8)	0.2744
Total bilirubin (mg/dl) (SD)	3.6 (±4.6)	3.8 (±5.2)	0.8023	4.4 (±5.6)	3.6 (±4.9)	0.2202
AST(IU/L) (SD)	428.5 (±409.7)	513.2 (±659.0)	0.5735	498.4 (±498.4)	467.1 (±550.9)	0.7813
ALT(IU/L) (SD)	488.1 (±492.3)	525.1 (±529.0)	0.7793	549.4 (±562.2)	502.2 (±505.8)	0.8568
ALP(IU/L) (SD)	450.3 (±185.0)	478.4 (±237.1)	0.6656	463.8 (±217.1)	464.0 (±212.0)	0.9474
IgG (mg/dl) (SD)	2587.1 (±1005.7)	2119.8 (±840.8)	2.10X10^-6^	2242.9 (±878.5)	2370.6 (±964.3)	0.3771
IgM(mg/dl) (SD)	216.5 (±283.5)	144.1 (±126.2)	0.0020	140.3 (±125.5)	185.0 (±230.7)	0.1716
Platelets (104/μl) (SD)	18.5 (±7.1)	18.5 (±7.5)	0.9008	18.1 (±6.6)	18.5 (±7.4)	0.8625
ANA ≧ 1:40, n (%)	164 (88.6%)	150 (85.7%)	*0.4328	29 (96.7%)	285 (86.4%)	*0.1510
ASMA ≧ 1:40, n (%)	91 (55.2%)	27 (19.1%)	*6.46X10^-11^	7 (33.3%)	111 (38.9%)	*0.6515
Cirrhosis, n (%)	26 (14.1%)	23 (13.1%)	*0.8782	8 (26.7%)	41 (12.4%)	*0.0462
IAIHG score (SD)	16.9 (3.1%)	15.6 (2.9%)	1.54X10^-5^	15.7 (2.9%)	16.3 (3.1%)	0.3476
Definite AIH, n (%)	133 (71.9%)	94 (53.7%)	*0.0005	17 (56.7%)	210 (63.6%)	*0.4383

Association was tested between AIH patients with or without DRB1*04:*05* or *13:*02* by Fisher's exact test using 2x2 contingency tables or Mann-Whitney's U test.

*Fisher's exact test was employed.

### *HLA-DRB1* genotype in type I AIH

We investigated the genotype frequency in the AIH patients (Table 4). The homozygosity for DRB1*04:*05* (OR 2.79, 95% CI 1.45–5.38) did not confer higher OR for AIH than heterozygosity for DRB1*04:*05* (OR 3.10, 95% CI 2.40–4.00). In contrast, the homozygosity for DRB1*13:*02* (OR 0.15, 95% CI 0.01–2.56) conferred lower OR than heterozygosity for DRB1*13:*02* (OR 0.51, 95% CI 0.34–0.78). The frequency of the DRB1*04:05/*13:*02* genotype was comparable. Of interest, higher frequencies of DRB1*04:05/*08:*02* (*P* = 3.78X10^-6^, OR 6.70, 95% CI 2.89–15.54) and DRB1*04:05/*08:*03* (*P* = 9.80X10^-7^, OR 4.54, 95% CI 2.47–8.35) genotypes in AIH were observed. Similarly, the DR4/DR8 genotype frequency in AIH was markedly increased (*P* = 3.12×10^−9^, OR 3.52, 95% CI 2.34–5.29). Thus, some specific heterozygous genotypes were predisposing for AIH.

**Table 4:** *HLA-DRB1* genotype frequency in the AIH patients and controls.

	Case (n = 360)	Control (n = 1026)	*P*	OR	95%CI
*04:*05*/not *04:*05*	167 (46.4)	224 (21.8)	5.72X10^-18^	3.10	(2.40–4.00)
*13:*02*/not *13:*02*	30 (8.3)	154 (15.0)	0.0011	0.51	(0.34–0.78)
*04:*01*/not *04:*01*	22 (6.1)	21 (2.0)	0.0003	3.11	(1.69–5.74)
*04:*01*/*04:*05*	4 (1.1)	1 (0.1)	0.0178	11.52	(1.28–103.39)
*04:*05*/*04:*05*	18 (5.0)	19 (1.9)	0.0035	2.79	(1.45–5.38)
*04:*05*/*08:*02*	18 (5.0)	8 (0.8)	3.78X10^-6^	6.70	(2.89–15.54)
*04:*05*/*08:*03*	27 (7.5)	18 (1.8)	9.80X10^-7^	4.54	(2.47–8.35)
*04:*05*/*13:*02*	8 (2.2)	25 (2.4)	1.0000	0.91	(0.41–2.04)
*13:*02*/*13:*02*	0 (0.0)	9 (0.9)	0.1225	0.15	(0.01–2.56)
DR4/DR4	34 (9.4)	57 (5.6)	0.0132	1.77	(1.14–2.76)
DR8/DR8	5 (1.4)	16 (1.6)	1.0000	0.89	(0.32–2.44)
DR6/DR6	3 (0.8)	25 (2.4)	0.0796	0.34	(0.10–1.12)
DR4/DR8	54 (15.0)	49 (4.8)	3.12X10^-9^	3.52	(2.34–5.29)
DR4/DR6	32 (8.9)	90 (8.8)	0.9144	1.01	(0.66–1.55)

AIH: autoimmune hepatitis, OR: odds ratio, 95%CI: confidence interval. Genotype frequencies are shown in parenthesis (%). Association was tested by Fisher's exact test using 2X2 contingency tables.

### Certain amino acid residues in HLA-DRβ chains were associated with AIH

**Fig 1:**
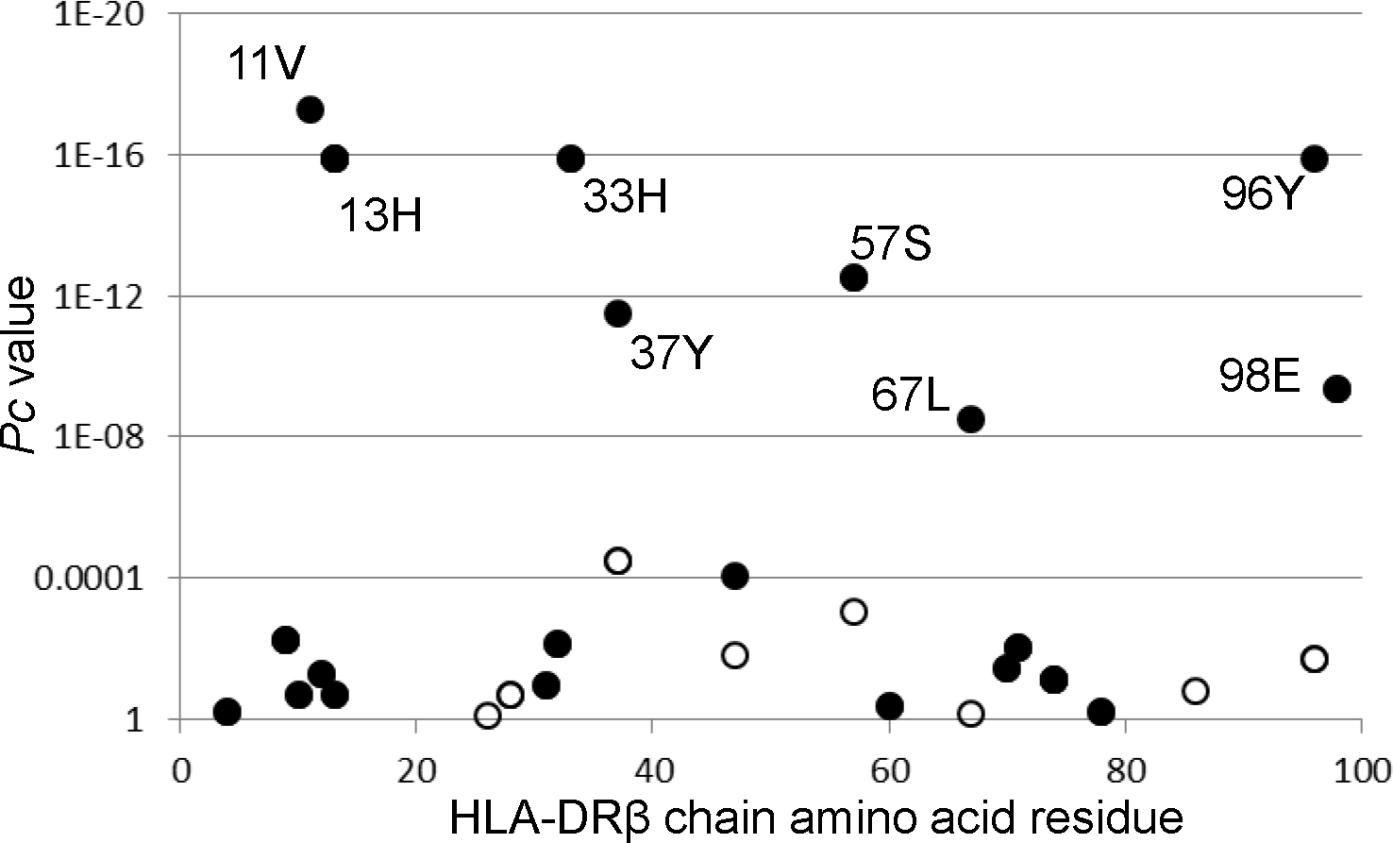
Associations of amino acid residues in DRβ chain with AIH. Each amino acid residue frequency in the HLA-DRβ chain for the 1026 healthy controls was compared with that of the AIH patients. Differences of amino acid residue carrier frequencies were analyzed by Fisher’s exact test using 2x2 contingency tables. Corrected *P* (*P*c) values were calculated by multiplying the *P* value by the number of amino acid residues tested. Predisposing associations were indicated by filled circles and protective associations by open circles.

The association with AIH with respect to each amino acid residue in the HLA-DRβ chain was analyzed. The amino acid residues of 11V, 13H, 33H, 57S, and 96Y in the DRβ chain showed associations with AIH (Fig 1). Thus, this association analysis suggested roles for specific amino acid residues in the HLA-DRβ chain.

### *HLA-DQB1* in type I AIH

We next tried to compare *HLA-DQB1* allele carrier frequency of the AIH patients with 413 of the 1026 healthy controls, since previously reported genotyping results of *DQB1* were available for the 413 healthy controls[24]. When *DRB1* genotyping results for the 413 healthy controls were compared with those of the AIH patients, similar tendencies were observed (S1 and S2 Tables). DQB1*04:*01* allele was strongly associated with AIH (*P* = 4.66×10^−18^, *Pc* = 6.99×10^−17^, OR 3.89, 95% CI 2.84–5.33, S3 Table). We further examined *HLA-DQB1* genotype (S4 Table). The homozygosity for DQB1*04:*01* (OR 4.04, 95% CI 1.48–11.08) conferred comparative OR compared with heterozygosity for DQB1*04:*01* (OR 3.47, 95% CI 2.52–4.77). The higher frequency of DQB1*04:01/*06:*01* genotype in AIH was observed (*P* = 8.75X10^-6^, OR 3.24, 95% CI 1.89–5.56). Thus, some of *DQB1*alleles or genotypes were predisposing for AIH.

### *DRB1-DQB1* haplotype in type I AIH

*DRB1-DQB1* haplotype carrier frequencies were compared between the AIH patients and the 413 healthy controls (Table 5). Higher carrier frequencies of DRB1*04:01-DQB1*03:*01* (*P* = 0.0007, OR 4.42 95% CI 1.77–11.01) and DRB1*04:05-DQB1*04:*01* (*P* = 1.99×10^−20^, OR 4.32, 95% CI 3.14–5.96) were found in the AIH patients. *DRB1-DQB1* diplotype frequencies were also compared between the AIH patients and the 413 healthy controls (Table 5). The homozygosity for DRB1*04:05-DQB1*04:*01* (OR 5.07, 95% CI 1.69–15.20) conferred slightly higher OR for AIH than heterozygosity for DRB1*04:05-DQB1*04:*01* (OR 3.81, 95% CI 2.76–5.28). The diplotype frequencies of DRB1*04:05-DQB1*04:01/DRB1*08:02-DQB1*03:*02* (*P* = 0.0004, OR 24.77, 95% CI 1.45–424.31) and DRB1*04:05-DQB1*04:01/DRB1*08:03-DQB1*06:*01* (*P* = 1.18×10^−6^, OR 10.64, 95% CI 3.19–35.46) were higher in AIH patients, suggesting the predisposing role of some specific heterozygous diplotypes in AIH.

**Table 5:** *DRB1-DQB1* haplotype carrier or diplotype frequency in the AIH patients and controls.

*DRB1-DQB*1 haplotype	Case (n = 360)	Control (n = 413)	*P*	OR	95%CI
*04:01-*03:*01*	22 (6.1)	6 (1.5)	0.0007	4.42	(1.77–11.01)
*04:05-*04:*01*	182 (50.6)	79 (19.1)	1.99X10^-20^	4.32	(3.14–5.96)
*08:02-*03:*02*	21 (5.8)	21 (5.1)	0.7508	1.16	(0.62–2.15)
*08:02-*04:*02*	13 (3.6)	16 (3.9)	1.0000	0.93	(0.44–1.96)
*08:03-*03:*01*	3 (0.8)	2 (0.5)	0.6682	1.73	(0.29–10.39)
*08:03-*06:*01*	57 (15.8)	57 (13.8)	0.4768	1.17	(0.79–1.75)
*13:02-*06:*04*	31 (8.6)	49 (11.9)	0.1559	0.70	(0.44–1.12)
*DRB1-DQB1* diplotype					
*0405-*0401/not *04:05-*04:*01*	165 (45.8)	75 (18.2)	1.17X10^-16^	3.81	(2.76–5.28)
*04:05-*04:*01*/*04:01-*03:*01*	4 (1.1)	1 (0.2)	0.1898	4.63	(0.52–41.61)
*04:05-*04:*01*/*04:05-*04:*01*	17 (4.7)	4 (1.0)	0.0015	5.07	(1.69–15.20)
*04:05-*04:*01*/*08:02-*03:*02*	10 (2.8)	0 (0.0)	0.0004	24.77	(1.45–424.31)
*04:05-*04:*01*/*08:02-*04:*02*	7 (1.9)	3 (0.7)	0.2017	2.71	(0.70–10.56)
*04:05-*04:*01*/*08:03-*03:*01*	1 (0.3)	0 (0.0)	0.4657	3.45	(0.14–84.97)
*04:05-*04:*01*/*08:03-*06:*01*	26 (7.2)	3 (0.7)	1.18X10^-6^	10.64	(3.19–35.46)
*04:05-*04:*01*/*13:02-*06:*04*	8 (2.2)	8 (1.9)	0.8052	1.15	(0.43–3.10)

AIH: autoimmune hepatitis, OR: odds ratio, 95%CI: confidence interval. Genotype frequencies are shown in parenthesis (%). Association was tested by Fisher's exact test using 2X2 contingency tables.

### Certain amino acid residues in HLA-DQβ chains were associated with AIH

**Fig 2:**
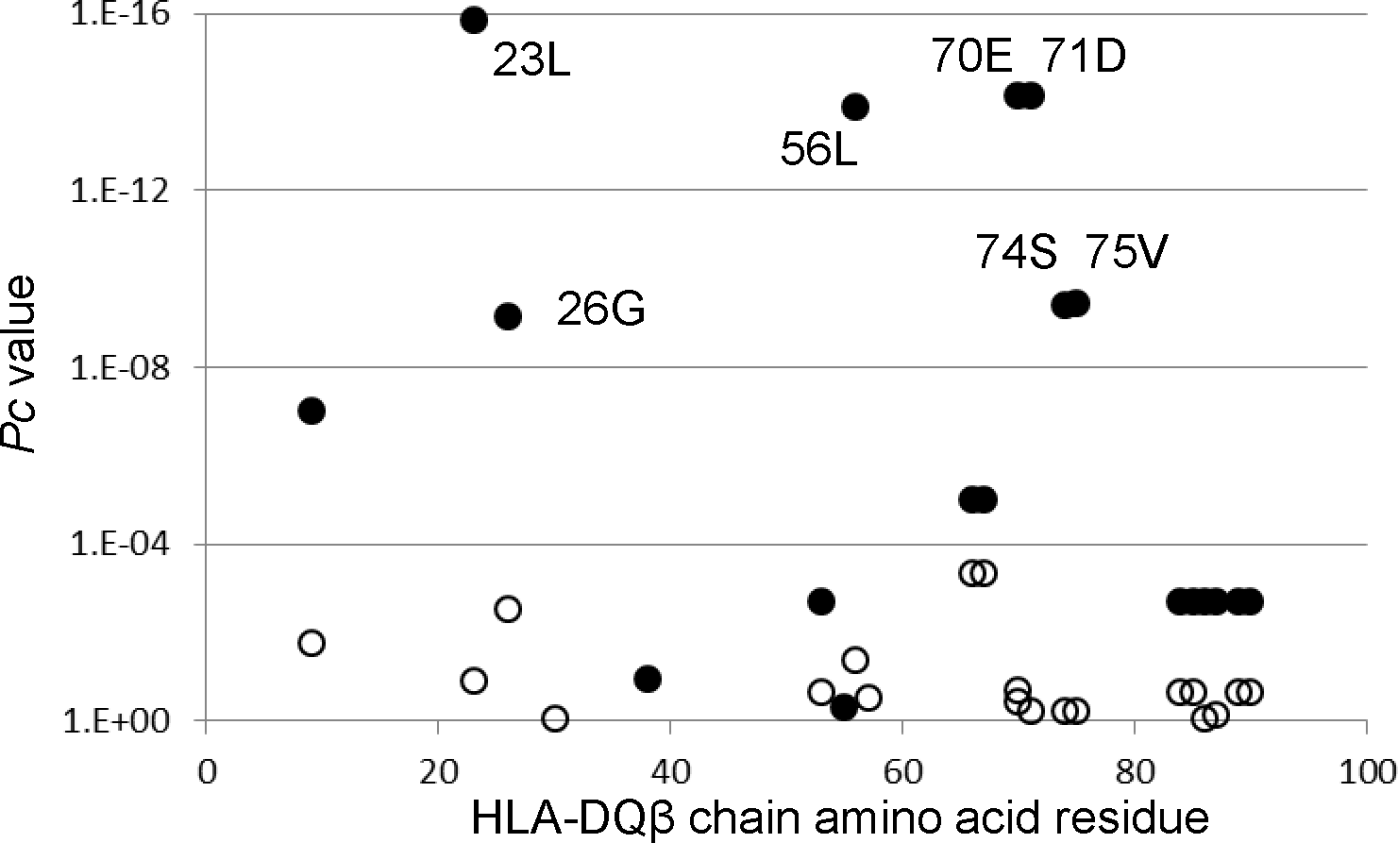
Associations of amino acid residues in DQβ chain with AIH. Each amino acid residue frequency in the HLA-DRβ chain for the 413 healthy controls was compared with that of the AIH patients. Differences of amino acid residue carrier frequencies were analyzed by Fisher’s exact test using 2x2 contingency tables. Corrected *P* (*P*c) values were calculated by multiplying the *P* value by the number of amino acid residues tested. Predisposing associations were indicated by filled circles and protective associations by open circles.

The association with AIH with respect to each amino acid residue in the HLA-DQβ chain was analyzed in the comparison with the 413 healthy controls. The amino acid residues of 23L, 56L, 70E, and 71D in the DQβ chain showed associations with AIH (Fig 2). When each amino acid residue frequency in the DRβ chain for the 413 healthy controls was compared with that of the AIH patients, similar tendencies were observed (S1 Fig). Thus, this association analysis suggested roles for specific amino acid residues in the HLA-DQβ chains.

## Discussion

**Fig 3:**
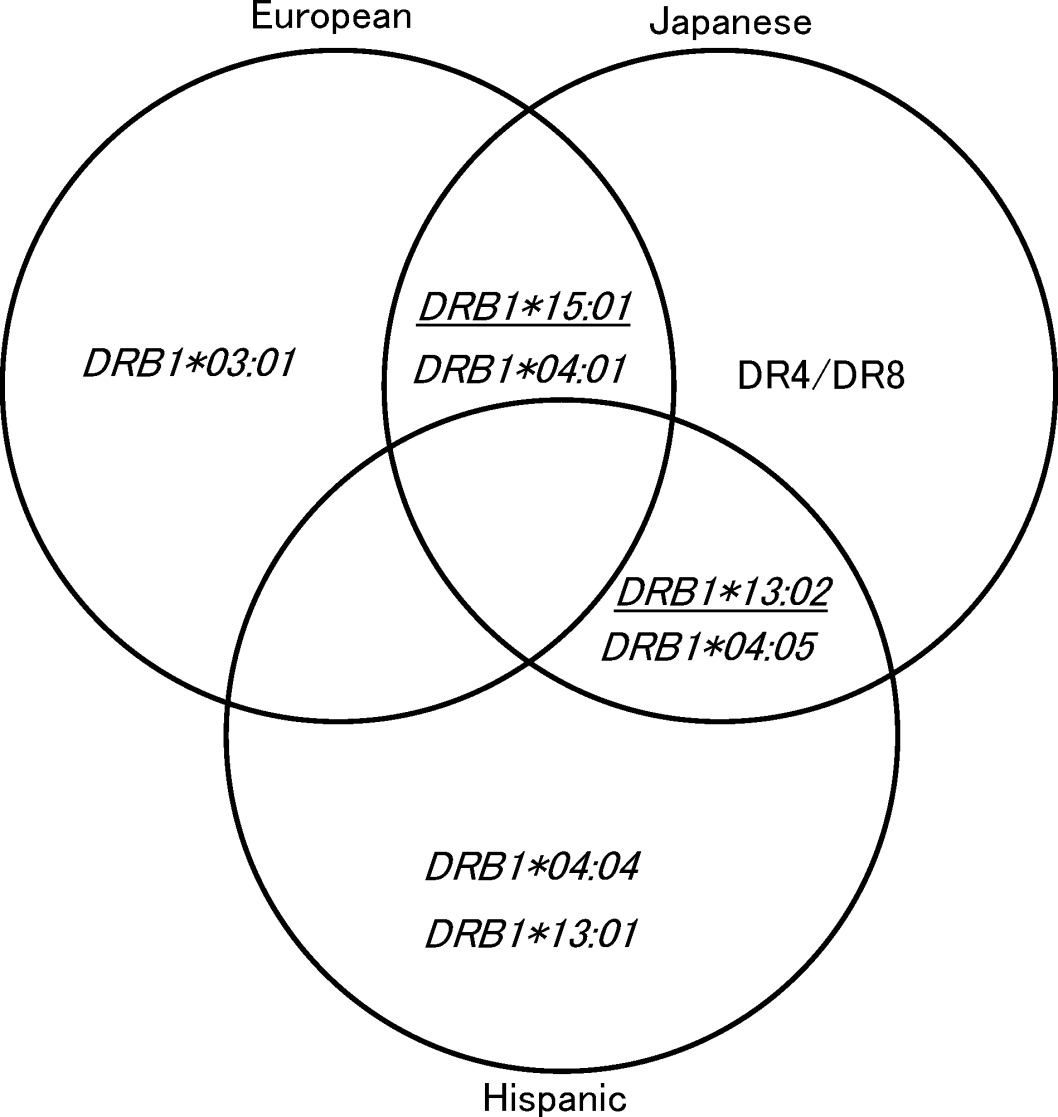
Summary of the *HLA-DRB1* alleles associated with type 1 AIH. The *HLA-DRB1* alleles associated with type 1 AIH in European, Japanese, and Hispanic populations are illustrated. The underlined alleles are protective alleles.

Several studies have reported that type 1 AIH is associated with HLA-DRB1*03:*01* and DRB1*04:*01* in European [5] and DRB1*04:*05* in Japanese populations (Fig 3) [6,7,8]. In the present study, we showed an association of Japanese AIH with DRB1*04:*01* and *04:*05*, indicating the common predisposing DRB1*04:*01* allele for AIH between European and Japanese populations. DRB1*04:*05* is also common predisposing allele for AIH between Latin America [13] and Japan. In previous studies, DRB1*13:*02* was protectively associated with type 1 AIH in Latin America [11,13,17]. We also confirmed a protective association of DRB1*13:*02* with Japanese AIH [8], but could not replicate the protective effects of DRB1*15:*01* [5,6]. These data indicated that the common protective DRB1*13:*02* allele for AIH between Latin America and Japan is also the protective allele shared by multiple autoimmune diseases [19].

Specific demographic features of Japanese AIH patients with DRB1*04:*05* were observed (Table 3). Elevated serum levels of IgG and IgM were detected in AIH patients with DRB1*04:*05*, as it was previously described [7]. In the present study, the IAIHG score, the positive rate of ASMA, and the rate of definite AIH were newly found to be higher in Japanese AIH patients with DRB1*04:*05*. Although *HLA* alleles are known to confer the risk for various autoimmune diseases, the precise mechanisms have not sufficiently been revealed. Thus, Japanese AIH patients with DRB1*04:*05* have typical clinical traits, probably because the auto-antigens presented by DRB1*04:05 molecules would be important for development of the typical clinical traits of AIH.

It is well known that DRB1*04:*01* in European [26,27] and DRB1*04:*05* in Japanese populations [23,28] are associated with the susceptibility for rheumatoid arthritis (RA) and type 1 diabetes, in an analogous fashion to AIH. RA is a systemic autoimmune disease that affects synovial joints. RA-susceptible *DRB1* alleles shared a conserved amino acid sequence at position 70–74 (QKRAA, RRRAA, or QRRAA) in HLA-DRβ chain and were designated as shared epitope alleles [23,26]. The shared epitope alleles include DRB1*01:*01*, *04:*01*, *04:*04*, *04:*05*, *04:*10*, *10:*01*, *14:*02*, and *14:*06*. However, neither DRB1*01:*01* nor *04:*10* seems to be a risk allele for AIH (Table 2). In the associations of *DRB1* alleles with susceptibility to RA, a gene dosage effect was reported; homozygosity for predisposing *DRB1* alleles confers higher OR than heterozygosity. However, we could not find any gene dosage effects of predisposing alleles or haplotypes in AIH. These data suggested the differential roles of *DRB1* in the pathogenesis between AIH and RA.

Type 1 diabetes is an autoimmune disease that affects pancreatic β cells producing insulin, resulting in the dysregulation of glucose metabolism. Susceptible *DRB1* alleles for type 1 diabetes are DRB1*03:*01*, *04:*01*, *04:*02*, *04:*04*, *04:*05*, and *08:*01* and protective alleles are DRB1*15:*01*, *14:*01*, and *07:*01* in European populations [27]. In Japanese populations, DRB1*04:*05*, *08:*02*, and *09:*01* are predisposing alleles for type 1 diabetes and DRB1*15:*02* is a protective allele [28]. No gene dosage effect was observed for DRB1*04:*05*, though a gene dosage effect for DRB1*09:*01* was detected for type 1 diabetes. Similarly, we did not detect any gene dosage effects of the predisposing DRB1*04:*05* allele in AIH (Table 4). In addition, higher frequencies of the DR3/DR4, DR4/DR4, and DR4/DR8 genotypes in type 1 diabetes were reported [27]. In an analogous fashion, frequencies of the DR4/DR8 genotypes were higher in Japanese type 1 AIH (Table 4). Since the allele frequency of DR4 is higher than that of DR8 in Japanese populations, DR4 is a risk allele by itself, but DR8 is not, and type 1 AIH is a multifactorial disease, the DR4/DR8 genotype could not mainly contribute to the pathogenesis of type 1 AIH. It was also reported that the association of DRB1*08 with the susceptibility of type 1 AIH was not detected in European populations [29], because of low frequency of DRB1*08. The DR4/DR8 heterozygous genotypes may cause an increased probability of self-antigen presentation, resulting in the increased risk of the diseases. Thus, the manner of *DRB1* association in type 1 AIH appears to be similar to that in type 1 diabetes.

Augmented frequencies of the DR3/DR4 and DR4/DR8 heterozygous genotypes in type 1 diabetes was explained by the pathologic importance of *trans*-complementing DQα-β heterodimer molecules encoded by the *DQA1* allele of one haplotype and the *DQB1* allele of the other haplotype. The low stability of these molecules in *trans* was proposed to be causative to type 1 diabetes [27,30]. Analogously, DR4/DR8 heterozygous genotype was increased in Japanese type 1 AIH (Table 4), suggesting that *trans*-complementing DQα-β heterodimer molecules might also play a role in AIH. *DRB1-DQB1* diplotype analysis revealed that DRB1*04:05-DQB1*04:01/DRB1*08:02-DQB1*03:*02* and DRB1*04:05-DQB1*04:01/DRB1*08:03-DQB1*06:*01* were significantly associated with AIH (Table 5). Japanese type 1 AIH was not significantly associated with DRB1*08:*02* or *08:*03* (Table 2). Neither DQB1*03:*02* nor *06:*01* was associated with type 1 AIH (S1 Table). Based on the conserved haplotype structure in the Japanese population, the *DQA1* allele in the haplotype of DRB1*04:05-DQB1*04:*01* is presumed to be DQA1*03:*03* [31] and the *DQA1* alleles in DRB1*08:02-DQB1*03:*02* and DRB1*08:03-DQB1*06:*01* are estimated to be DQA1*03:*01* and *01:*03*, respectively. The high risk diplotype DRB1*04:05-DQB1*04:01/DRB1*08:02-DQB1*03:*02* is considered to encode DQA1*03:03-DQB1*04:01 and DQA1*03:01-DQB1*03:02 molecules in *cis* (DQα-β heterodimer molecules formed by the protein products of *DQA1* and the *DQB1* alleles from the same chromosome) and DQA1*03:03-DQB1*03:02 and DQA1*03:01-DQB1*04:01 molecules in *trans* (DQα-β heterodimer molecules formed by the protein products of *DQA1* and the *DQB1* alleles from the opposite chromosomes). The stabilities of these four types of DQα-β heterodimer molecules were estimated to be low, according to the previous study [30]. The other high risk diplotype DRB1*04:05-DQB1*04:01/DRB1*08:03-DQB1*06:*01* is thought to encode DQA1*03:03-DQB1*04:01 and DQA1*01:03-DQB1*06:01 molecules in *cis* and DQA1*03:03-DQB1*06:01 and DQA1*01:03-DQB1*04:01 molecules in *trans*. The stabilities of these molecules except DQA1*01:03-DQB1*06:01 in *cis* were also estimated to be low [30]. In patients with the risk diplotype DRB1*04:05-DQB1*04:01/DRB1*08:03-DQB1*06:*01*, the low stability of *trans*-complementing DQα-β heterodimer molecules could explain the pathogenesis of type 1 AIH. In the case of type 1 diabetes, each of *DRB1*, *DQA1*, and *DQB1* is believed to have independent genetic contribution in the disease susceptibility based on the data from haplotype analysis [27]. Similar scenario might also apply to AIH. However, such analysis could not be performed in this study, because of the limited variety of *DRB1*-*DQB1* haplotypes in Japanese populations (Table 5). Furthermore, other culprit genes in linkage disequilibrium with *DRB1-DQB1* loci might be causative for AIH. Thus, the results of the association analyses of *DRB1* and *DQB1* in type 1 AIH could propose several lines of explanations on the mechanisms underlined in the pathogenesis.

We detected that amino acid residues of 11V, 13H, 33H, 57S, and 96Y in the HLA-DRβ chain were associated with AIH (Fig 1A); these amino acids were encoded by DRB1*04:*05* allele. It was also found that some amino acid residues of the DQβ chains were associated with type 1 AIH (Fig 1B). These amino acid residues were also encoded by DQB1*04:*01*. These data were influenced by the strongest predisposing haplotype DRB1*04:05-DQB1*04:*01* for AIH, confirming the dominance of the DRB1*04:05-DQB1*04:*01* haplotype in type 1 AIH in Japanese populations.

In conclusion, we showed the predisposing association of DRB1*04:*01*, DRB1*04:*05*, and DQB1*04:*01* and the protective association of DRB1*13:*02* with Japanese type 1 AIH. The association of DR4/DR8 heterozygous genotype with AIH was newly noted. With respect to *DRB1-DQB1* haplotypes, DRB1*04:01-DQB1*03:*01* and DRB1*04:05-DQB1*04:*01* haplotypes were found to be associated with type 1 AIH. Of interest, the association of DRB1*04:05-DQB1*04:01/DRB1*08:02-DQB1*03:*02* and DRB1*04:05-DQB1*04:01/DRB1*08:03-DQB1*06:*01* diplotypes was revealed. These data suggested the roles of specific combinations of *DRB1* and *DQB1* alleles or haplotypes in the pathogenesis of type 1 AIH. Further large scale studies should be performed to confirm these findings. In addition, because the *HLA* allele distribution pattern is different in other ethnic populations, it would be intriguing and informative to analyze *DRB1* and *DQB1* alleles in type 1 AIH in other populations.

## Supporting information

S1 Fig Associations of amino acid residues in DRβ chain with AIH. (PDF)

S1 Table *HLA-DRB1* allele carrier frequency in the AIH patients and the 413healthy controls. (PDF)

S2 Table *HLA-DRB1* genotype frequency in the AIH patients and the 413 healthy controls. (PDF)

S3 Table *HLA-DQB1* allele carrier frequency in the AIHpatients and the 413 healthy controls. (PDF)

S4 Table *HLA-DQB1* genotype frequency in the AIH patients andthe 413 healthy controls. (PDF)
