# Supplementary material for: HLA-DRB1 and DQB1 alleles in Japanese type 1 autoimmune hepatitis: The predisposing role of the DR4/DR8 heterozygous genotype
Source: PLoS One. 2017 Oct 31;12(10):e0187325. doi: 10.1371/journal.pone.0187325 (PMC5663488; doi:10.1371/journal.pone.0187325)
Supplement: S1 Fig — (PDF) [file pone.0187325.s001.pdf]

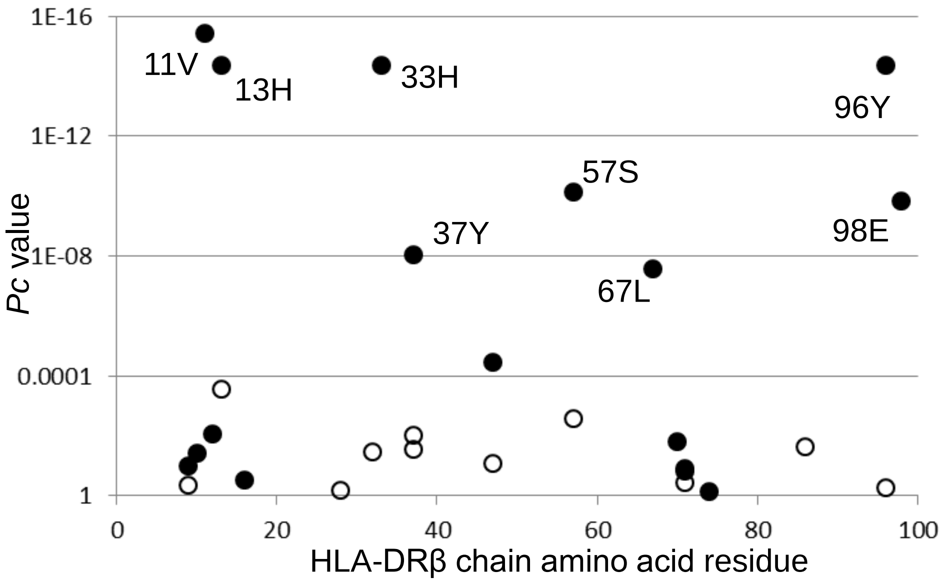

Supplementary Figure S1. Associations of amino acid residues in DRβ chain with AIH. Each amino acid residue frequency in the HLA-DRβ chain for the 413 healthy controls was compared with that of the AIH patients. Differences of amino acid residue carrier frequencies were analyzed by Fisher's exact test using 2x2 contingency tables. Corrected *P* (*P*c) values were calculated by multiplying the *P* value by the number of amino acid residues tested. Predisposing associations were indicated by filled circles and protective associations by open circles.
