# Supplementary material for: HLA-DRB1 and DQB1 alleles in Japanese type 1 autoimmune hepatitis: The predisposing role of the DR4/DR8 heterozygous genotype
Source: PLoS One. 2017 Oct 31;12(10):e0187325. doi: 10.1371/journal.pone.0187325 (PMC5663488; doi:10.1371/journal.pone.0187325)
Supplement: S1 Table — (PDF) [file pone.0187325.s002.pdf]

Supplementary Table S1. *HLA-DRB1* allele carrier frequency in the AIH patients and the 413 healthy controls.

	Case (n=360)	Control (n=413)	*P*	OR	Pc	95%CI
*DRB1*01:01*	26 (7.2)	42 (10.2)	0.1627	0.69	NS	(0.41-1.15)
*DRB1*03:01*	1 (0.3)	2 (0.5)	1.0000	0.57	NS	(0.05-6.34)
*DRB1*04:01*	22 (6.1)	7 (1.7)	0.0019	3.78	0.0544	(1.59-8.95)
*DRB1*04:03*	15 (4.2)	19 (4.6)	0.8611	0.90	NS	(0.45-1.80)
*DRB1*04:05*	185 (51.4)	87 (21.1)	$9.26X10^{-19}$	3.96	$2.69X10^{-17}$	(2.89-5.42)
*DRB1*04:06*	15 (4.2)	34 (8.2)	0.0257	0.48	0.7460	(0.26-0.91)
*DRB1*04:07*	5 (1.4)	3 (0.7)	0.4831	1.92	NS	(0.46-8.11)
*DRB1*04:10*	12 (3.3)	14 (3.4)	1.0000	0.98	NS	(0.45-2.15)
*DRB1*07:01*	2 (0.6)	3 (0.7)	1.0000	0.76	NS	(0.13-4.60)
*DRB1*08:02*	35 (9.7)	38 (9.2)	0.8067	1.06	NS	(0.66-1.72)
*DRB1*08:03*	58 (16.1)	61 (14.8)	0.6187	1.11	NS	(0.75-1.64)
*DRB1*08:09*	0 (0.0)	1 (0.2)	1.0000	0.38	NS	(0.02-9.39)
*DRB1*09:01*	77 (21.4)	105 (25.4)	0.2028	0.80	NS	(0.57-1.12)
*DRB1*10:01*	5 (1.4)	2 (0.5)	0.2604	2.89	NS	(0.56-15.01)
*DRB1*11:01*	8 (2.2)	22 (5.3)	0.0384	0.40	NS	(0.18-0.92)
*DRB1*12:01*	25 (6.9)	29 (7.0)	1.0000	0.99	NS	(0.57-1.72)
*DRB1*12:02*	11 (3.1)	10 (2.4)	0.6603	1.27	NS	(0.53-3.03)
*DRB1*13:01*	2 (0.6)	5 (1.2)	0.4587	0.46	NS	(0.09-2.36)
*DRB1*13:02*	30 (8.3)	57 (13.8)	0.0168	0.57	0.4870	(0.36-0.91)
*DRB1*14:02*	1 (0.3)	0 (0.0)	0.4657	3.45	NS	(0.14-84.97)
*DRB1*14:03*	5 (1.4)	21 (5.1)	0.0046	0.26	0.1326	(0.10-0.70)
*DRB1*14:04*	0 (0.0)	1 (0.2)	1.0000	0.38	NS	(0.02-9.39)
*DRB1*14:05*	13 (3.6)	14 (3.4)	1.0000	1.07	NS	(0.50-2.30)
*DRB1*14:06*	5 (1.4)	16 (3.9)	0.0444	0.35	NS	(0.13-0.96)
*DRB1*14:07*	1 (0.3)	1 (0.2)	1.0000	1.15	NS	(0.07-18.42)
*DRB1*14:54*	20 (5.6)	28 (6.8)	0.5511	0.81	NS	(0.45-1.46)
*DRB1*15:01*	41 (11.4)	68 (16.5)	0.0489	0.65	NS	(0.43-0.99)
*DRB1*15:02*	62 (17.2)	89 (21.5)	0.1457	0.76	NS	(0.53-1.09)
*DRB1*16:02*	5 (1.4)	5 (1.2)	1.0000	1.15	NS	(0.33-4.00)
DR4	248 (68.9)	150 (36.3)	$9.91X10^{-20}$	3.88		(2.88-5.24)
DR6 (**13, *14*)	67 (18.6)	137 (33.2)	$4.37X10^{-6}$	0.46		(0.33-0.64)
DR8	91 (25.3)	95 (23.0)	0.5000	1.13		(0.81-1.58)

AIH: autoimmune hepatitis, OR: odds ratio, CI: confidence interval, Pc: corrected P value, NS: not significant. Allele carrier frequencies are shown in parenthesis (%). Association was tested by Fisher's exact test using 2x2 contingency tables under the dominant model.
