# Supplementary material for: HLA-DRB1 and DQB1 alleles in Japanese type 1 autoimmune hepatitis: The predisposing role of the DR4/DR8 heterozygous genotype
Source: PLoS One. 2017 Oct 31;12(10):e0187325. doi: 10.1371/journal.pone.0187325 (PMC5663488; doi:10.1371/journal.pone.0187325)
Supplement: S2 Table — (PDF) [file pone.0187325.s003.pdf]

Supplementary Table S2. *HLA-DRB1* genotype frequency in the AIH patients and the 413 healthy controls.

	Case (n=360)	Control (n=413)	*P*	OR	95%CI
**04:05*/not **04:05*	167 (46.4)	80 (19.4)	$8.50X10^{-16}$	3.60	(2.62-4.96)
**13:02*/not **13:02*	30 (8.3)	52 (12.6)	0.0612	0.63	(0.39-1.01)
**04:01*/not **04:01*	22 (6.1)	6 (1.5)	0.0007	4.42	(1.77-11.01)
**04:01/*04:05*	4 (1.1)	1 (0.2)	0.1898	4.63	(0.52-41.61)
**04:05/*04:05*	18 (5.0)	7 (1.7)	0.0131	3.05	(1.26-7.40)
**04:05/*08:02*	18 (5.0)	4 (1.0)	0.0008	5.38	(1.80-16.05)
**04:05/*08:03*	27 (7.5)	4 (1.0)	$3.03X10^{-6}$	8.29	(2.87-23.93)
**04:05/*13:02*	8 (2.2)	8 (1.9)	0.8052	1.15	(0.43-3.10)
**13:02/*13:02*	0 (0.0)	5 (1.2)	0.0646	0.10	(0.01-1.87)
DR4/DR4	34 (9.4)	22 (5.3)	0.0362	1.85	(1.06-3.23)
DR8/DR8	5 (1.4)	8 (1.9)	0.5897	0.71	(0.23-2.20)
DR6/DR6	3 (0.8)	11 (2.7)	0.0632	0.31	(0.08-1.11)
DR4/DR8	54 (15.0)	16 (3.9)	$6.17X10^{-8}$	4.38	(2.46-7.80)
DR4/DR6	32 (8.9)	32 (7.7)	0.6020	1.16	(0.70-1.94)

AIH: autoimmune hepatitis, OR: odds ratio, 95%CI: confidence interval. Genotype frequencies are shown in parenthesis (%). Association was tested by Fisher's exact test using 2X2 contingency tables.
