# Supplementary material for: HLA-DRB1 and DQB1 alleles in Japanese type 1 autoimmune hepatitis: The predisposing role of the DR4/DR8 heterozygous genotype
Source: PLoS One. 2017 Oct 31;12(10):e0187325. doi: 10.1371/journal.pone.0187325 (PMC5663488; doi:10.1371/journal.pone.0187325)
Supplement: S3 Table — (PDF) [file pone.0187325.s004.pdf]

Supplementary Table S3. *HLA-DQB1* allele carrier frequency in the AIH patients and the 413 healthy controls.

	Case (n=360)	Control (n=413)	*P*	OR	*P*c	95%CI
*DQB1*02:01*	1 (0.3)	4 (1.0)	0.3793	0.28	NS	(0.03-2.56)
*DQB1*03:01*	74 (20.6)	96 (23.2)	0.3849	0.85	NS	(0.61-1.20)
*DQB1*03:02*	57 (15.8)	83 (20.1)	0.1346	0.75	NS	(0.52-1.08)
*DQB1*03:03*	80 (22.2)	111 (26.9)	0.1552	0.78	NS	(0.56-1.08)
*DQB1*03:06*	1 (0.3)	0 (0.0)	0.4657	3.45	NS	(0.14-84.97)
*DQB1*04:01*	182 (50.6)	86 (20.8)	$4.66X10^{-18}$	3.89	$6.99X10^{-17}$	(2.84-5.33)
*DQB1*04:02*	26 (7.2)	29 (7.0)	1.0000	1.03	NS	(0.60-1.79)
*DQB1*05:01*	31 (8.6)	44 (10.7)	0.3941	0.79	NS	(0.49-1.28)
*DQB1*05:02*	16 (4.4)	16 (3.9)	0.7204	1.15	NS	(0.57-2.34)
*DQB1*05:03*	23 (6.4)	34 (8.2)	0.3384	0.76	NS	(0.44-1.32)
*DQB1*06:01*	117 (32.5)	144 (34.9)	0.4937	0.90	NS	(0.67-1.21)
*DQB1*06:02*	38 (10.6)	65 (15.7)	0.0434	0.63	0.6516	(0.41-0.97)
*DQB1*06:03*	2 (0.6)	6 (1.5)	0.2958	0.38	NS	(0.08-1.89)
*DQB1*06:04*	30 (8.3)	50 (12.1)	0.0976	0.66	NS	(0.41-1.06)
*DQB1*06:09*	0 (0.0)	6 (1.5)	0.0328	0.09	0.4927	(0.00-1.55)

AIH: autoimmune hepatitis, OR: odds ratio, CI: confidence interval, Pc: corrected P value, NS: not significant. Allele carrier frequencies are shown in parenthesis (%). Association was tested by Fisher's exact test using 2X2 contingency tables under the dominant model.
