# Supplementary material for: HLA-DRB1 and DQB1 alleles in Japanese type 1 autoimmune hepatitis: The predisposing role of the DR4/DR8 heterozygous genotype
Source: PLoS One. 2017 Oct 31;12(10):e0187325. doi: 10.1371/journal.pone.0187325 (PMC5663488; doi:10.1371/journal.pone.0187325)
Supplement: S4 Table — (PDF) [file pone.0187325.s005.pdf]

SupplementaryTable S4. *HLA-DQB1* genotype frequency in the AIH patients and the 413 healthy controls.

	Case (n=360)	Control (n=413)	*P*	OR	95%CI
**04:01*/not **04:01*	165 (45.8)	81 (19.6)	$5.50X10^{-15}$	3.47	(2.52-4.77)
**04:01*/**04:01*	17 (4.7)	5 (1.2)	0.0041	4.04	(1.48-11.08)
**04:01*/**03:01*	24 (6.7)	9 (2.2)	0.0022	3.21	(1.47-6.99)
**04:01*/**03:02*	17 (4.7)	9 (2.2)	0.0702	2.22	(0.98-5.05)
**04:01*/**04:02*	8 (2.2)	4 (1.0)	0.2429	2.32	(0.69-7.78)
**04:01*/**06:01*	51 (14.2)	20 (4.8)	$8.75X10^{-6}$	3.24	(1.89-5.56)

AIH: autoimmune hepatitis, OR: odds ratio, 95%CI: confidence interval. Genotype frequencies are shown in parenthesis (%). Association was tested by Fisher's exact test using 2X2 contingency tables.
